# Ultrasound-guided percutaneous transhepatic one-step biliary fistulation combined with rigid choledochoscopy for recurrent hepatolithiasis

**DOI:** 10.1186/s12893-024-02370-x

**Published:** 2024-03-27

**Authors:** Wu Pan, Yuhang Lu, JunJie Li, Jia Zhang, Shenglin Chen

**Affiliations:** 1 Department of Hepatobiliary Surgery, Heyou Hospital, No. 1 of Heren Road, Junlan Community, Beijiao Town, Foshan City, Guangdong Province 528306 China; 2 Department of General Surgery, The Second Affiliated Hospital of Nanchang University, Nanchang, Jiangxi Province 330000 China; 3 Department of Hepatobiliary Surgery, Wuhu Hospital Affiliated to East China Normal University, Wuhu, Anhui Province 241000 China

**Keywords:** Hepatolithiasis, Percutaneous transhepatic choledochoscopy, Reoperation

## Abstract

**Purpose:**

Percutaneous transhepatic one-step biliary fistulation (PTOBF) is used to treat choledocholithiasis and biliary stricture. This study aimed to evaluate the safety and efficacy of ultrasound-guided PTOBF combined with rigid choledochoscopy in the treatment of recurrent hepatolithiasis.

**Materials and methods:**

The clinical data of 37 consecutive patients who underwent PTOBF combined with rigid choledochoscopy for RHL from March 2020 to March 2022 at our hospital were retrospectively analyzed.

**Results:**

A total of 68 percutaneous transhepatic punctures were performed in 37 patients, with a puncture success rate of 85.29% (58/68) and a dilatation success rate of 100.00% (58/58). The mean blood loss of operation was 9.84 ± 18.10 mL, the mean operation time was 82.05 ± 31.92 min, and the mean length of postoperative hospital stay was 5.59 ± 3.26 days. The initial stone clearance rate was 40.54% (15/37) and the final stone clearance rate was 100% (37/37). The incidence of postoperative complications was 10.81% (4/37), including 2 cases of pleural effusion, 1 case of hemorrhage, and 1 case of cholangitis, which recovered after treatment. During a mean follow-up period of 23 months (range 12 to 36 months), only 1 patient experienced stone recurrence.

**Conclusion:**

Ultrasound-guided PTOBF combined with rigid choledochoscopy in the treatment of RHL based on skilful manipulation seems to be a safe, effective and minimally invasive method with clinical application value. Further comparative studies with large sample sizes are needed in the future to confirm the reliability of its therapeutic results.

## Introduction

Hepatolithiasis (HL) is very common in East Asia, with an incidence of 30% -50%, while in Western countries, the number is only 0.6% -1.3% [1, 2]. In recent years, with the changes in economic conditions as well as dietary habits, the incidence of HL in Eastern countries has declined, while the incidence in Western countries is gradually increasing with the increase of Asian immigrants [1, 3]. The mechanism of HL formation remains to be further elucidated and is generally considered closely related to infection, bile duct stenosis, and anatomical abnormalities [3, 4].

Recurrent hepatolithiasis (RHL) refers to the reappearance of HL after surgery and is the most common postoperative complication of HL, with an incidence of approximately 4–24% [5, 6]. Surgery is the mainstay of treatment for HL and RHL, with the primary therapeutic goals being stone removal and prevention of recurrence [7, 8]. Although many mature methods have been used to treat RHL, there are still high rates of stone retention, recurrence, and complication rates after surgery [5, 9, 10]. In patients with RHL, reoperation is often difficult due to a history of previous abdominal surgery that may have resulted in abdominal adhesions and insufficient liver volume.

In 1974, Japanese scholars first reported the use of percutaneous transhepatic cholangioscopy (PTCS) for the treatment of HL with good results [11]. PTCS avoids reoperation while preserving the hepatic parenchyma, and is particularly suitable for patients with RHL who have insufficient hepatic reserve and stones in multiple bile ducts [12, 13]. Compared with conventional PTCS, which requires multiple sinus expansion, modified PTOBF based on PTCS combined with rigid choledochoscopy can expand the sinus in a single procedure, which provides a shorter treatment period and better treatment outcomes, thus gradually being applied in clinical practice [12–14].

There is no consensus on the reoperation plan for RHL and there is a lack of reports. In this study, we analyzed the experience of PTOBF in the treatment of RHL to evaluate the safety and efficacy of this technique in the treatment of RHL.

## Methods

### Ethics

The study was approved by the Ethics Committee of the Wuhu Hospital Affiliated to East China Normal University, written informed consent was obtained from each patient, and all data were used only for scientific research.

### Patients

From March 2020 to March 2022, 37 consecutive patients in the Wuhu Hospital Affiliated to East China Normal University received PTOBF combined with rigid cholangioscopy for RHL, and we retrospectively analyzed the clinical data of these patients.

Inclusion criteria were as follows: (1) meets the diagnosis of RHL (previous surgery for hepatolithiasis, reoccurrence of hepatolithiasis); (2) preoperative liver function assessments were Child–Pugh A or B grades; (3) dilated intrahepatic bile ducts (≥ 3 mm), without significant fibrosis or atrophy of the liver parenchyma; (4) underwent PTOBF combined rigid cholangioscopy treatment. Exclusion criteria were as follows: (1) incomplete clinical data; (2) Combined benign or malignant tumours of the liver; (3) Combined organic damage and dysfunction of important organs, unable to tolerate general anaesthesia surgery (4) Extra-hepatic bile ducts with severe stenosis or inflammation, requiring biliary-intestinal anastomosis.

All patients had undergone at least one surgery for HL. Preoperatively, we performed a complete evaluation of each patient, including serology, electrocardiogram, chest X-ray, ultrasound (US), computed tomography (CT), magnetic resonance imaging (MRI), and magnetic resonance cholangiopancreatography (MRCP), to confirm the patient’s diagnosis of RHL. Preoperative imaging helped us to determine the location, size, and number of stones and helped us to understand the anatomy of the patient’s biliary and vascular systems so that we could plan for puncture before surgery.

### PTOBF procedure

All surgeries were completed by the author’s team and were given the same postoperative treatment.

**Fig. 1:**
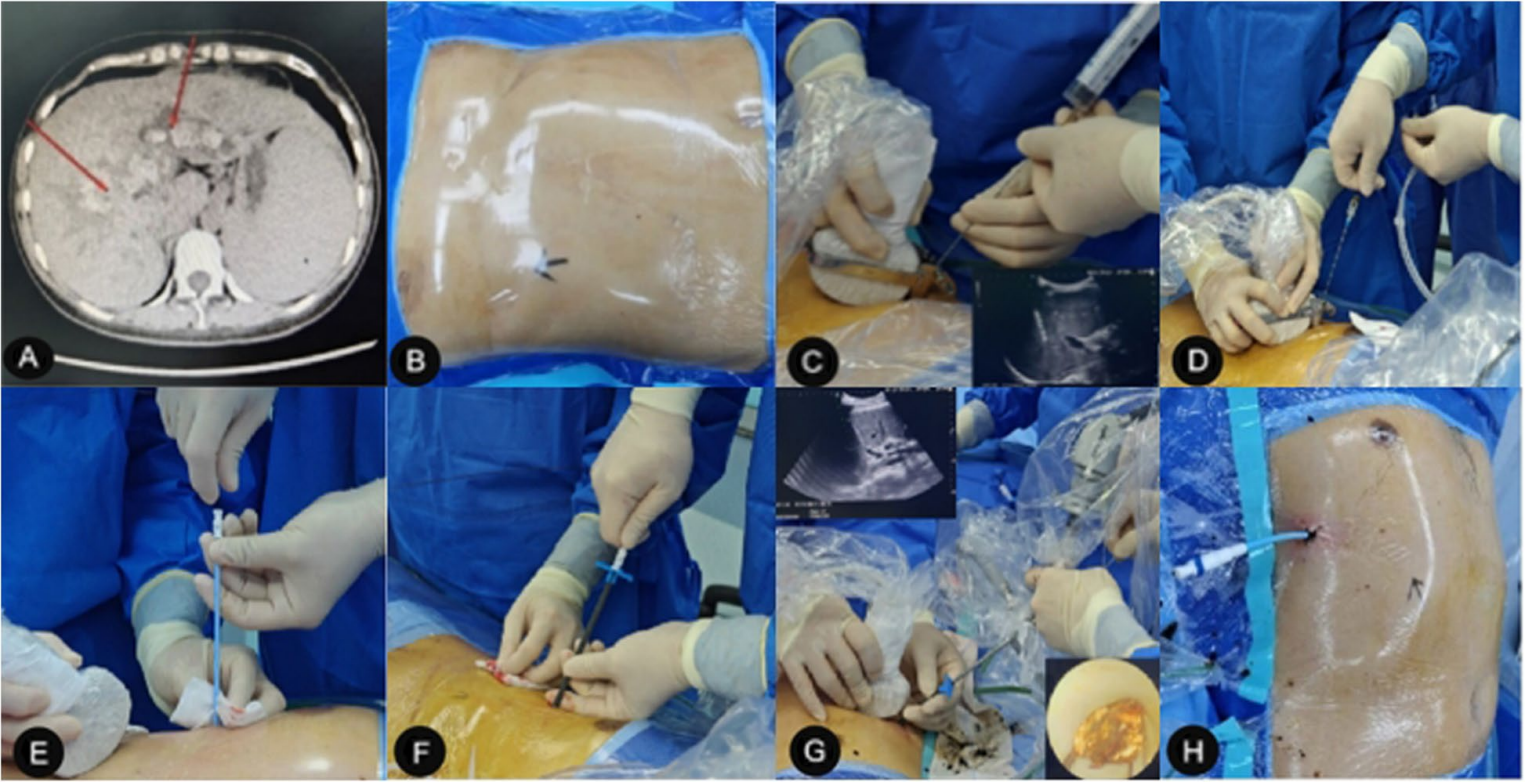
PTOBF surgical procedure. **A** Puncture plan based on preoperative imaging findings. **B** Intraoperative US exploration to determine the puncture point. **C** After US-guided puncture, bile was seen on retraction to represent successful puncture. **D** US-assisted judgment of guidewire placement position. **E** US-guided dilator gradually dilates the sinus. **F** Placement of 14Fr protective sheath. **G** US-assisted choledochoscopic stone extraction. **H** Placement of a “J” shaped drain

1. The patients were placed in the supine position, with general anesthesia by endotracheal intubation, and the right side of the back was slightly elevated (5 cm) to facilitate puncture. According to the intraoperative US exploration results, combined with preoperative CT and MRI/MRCP images, the appropriate puncture point and puncture path were selected (Fig. 1A, B).
2. US-guided percutaneous transhepatic puncture of the puncture needle into the target bile duct. The puncture path can be fully visualized by US. After the puncture needle reaches the target bile duct, the needle core is withdrawn from the needle and the bile is withdrawn by a syringe, which means that the puncture is successful (Fig. 1C). If a bloody fluid is seen on syringe retraction, the needle may have entered the vessel, and it is sufficient to reinsert the needle core and slowly withdraw the needle.
3. Place the guidewire inside the puncture needle, and at the same time, use US to make sure that the guidewire has entered the bile duct, then withdraw the puncture needle (Fig. 1D).
4. Measure the length of the needle into the skin and make a small incision of approximately 5 mm at the puncture point. A short pause for breathing (no more than 2 min) may be used to start the puncture to avoid the position of the liver changing with breathing during the puncture. A biliary expander of 8Fr, 10Fr, 12Fr, and 14Fr is inserted sequentially along the guidewire to dilate the sinusoids (Fig. 1E). The 14Fr dilator with a protective sheath is then inserted into the sinus, taking care that the length should be the same as the length of the puncture needle into the skin. At this point, the position of the expander can be determined by US (Fig. 1F).
5. The dilator is withdrawn, and the sheath continues to be placed in the sinus as a channel between the bile duct and the outside of the body. A rigid choledochoscope (F8/9.8 cm; Richard Wolf GmbH, Germany) is placed for stone extraction or lithotripsy. Under choledochoscope surveillance, small stones can be flushed out with the water flow, large stones can be removed with a mesh basket or forceps, and harder stones can be fragmented with a lithotripter (pneumatic lithotripter, type JML-6; Shenzhen Juxinglong Medical Equipment Co., Ltd., Guangdong Province, China). US assists in determining the location of the bile ducts and stones, and guides the choledochoscope to the location of the stone, which can help to retrieve the stone (Fig. 1G).
6. If a single channel cannot remove the stone, multiple channels can be established as appropriate, and the number of channels usually does not exceed 4. Postoperatively, a 14Fr “J” shaped external drain is left in the bile duct for postoperative cholangiography, bile duct decompression, support of the narrowed bile duct (intrahepatic and extrahepatic), and second-stage stone extraction or biliary exploration (After 6 to 8 weeks of surgery, when the percutaneous transhepatic sinusoidal tract has completely formed, and the patient’s hepatic function and physical condition have recovered from a period of biliary decompression and drainage, there is a lower risk of performing a repeat stone extraction. At this time, it is relatively safe to remove the external drain if it is confirmed that no stones are remaining) (Fig. 1H).

### Follow-up

Beginning one month after surgery, each patient was followed up in the outpatient department every 3 months until April 2023. The follow-up includes US, CT, MRI, MRCP, and serological examination to record stone clearance and complications. HL detected within 6 months after surgery was defined as residual stone, and HL detected after 6 months after surgery was defined as stone recurrence.

### Statistical analysis

Data were retrospectively analyzed for patient characteristics, perioperative outcomes, and follow-up outcomes. Quantitative data were presented as mean ± standard deviation (SD), and qualitative data were presented as rate (%).

## Results

### Patient characteristics

The mean age of the patients was 52.78 ± 15.48 and included 14 males (37.8%) and 23 females (62.2%). All patients had a history of biliary surgery with a per capita number of 2.11 ± 1.41, and PTOBF was successfully performed in all patients. The distribution of stones in the patients is shown in Table 1.

**Table 1:** Patient characteristics

Variables	Value (*n* = 37)
Gender (male: female)	14:23
Age (years), mean ± SD	52.78 ± 15.48
Child–Pugh (A: B)	31:6
Previous biliary surgery, n (%)	
Hepatectomy	13 (35.14%)
Cholangiojejunostomy	9 (24.32%)
Traditional PTCS	7 (18.92%)
Choledocholithotomy	23 (62.16%)
Cholecystectomy	36 (97.30%)
Stone Location, n (%)	
Left hepatic	13 (35.14%)
Right hepatic	10 (27.03%)
Bilatera hepatic	14 (37.84%)
CBDS, yes. (%)	8 (21.62%)
Biliary Stricture, yes. (%)	15 (40.54%)

*CBDS* common bile duct stone

### Perioperative outcomes

A total of 68 punctures were performed in 37 patients with a puncture success rate of 85.29% (58/68) and a dilatation success rate of 100.00% (58/58). The mean intraoperative blood loss was 9.84 ± 18.10 mL, the mean operative time was 82.05 ± 31.92 min, and the mean postoperative hospitalization was 5.59 ± 3.26 days. Two cases of pleural effusion, one case of biliary hemorrhage, and one case of cholangitis occurred in the postoperative period. Preoperative and postoperative liver function and enzyme changes are shown in Table 2. Two patients with pleural effusion improved after puncture drainage and albumin supplementation, and the rest improved after conservative treatment (Table 2).

**Table 2:** Perioperative outcomes

Variables	Value (*n* = 37)
Puncture (success: total)	58:68
Dilatation (success: total)	58:58
Intraoperative blood loss (ml), mean ± SD	9.84 ± 18.10
Operation time (min), mean ± SD	82.05 ± 31.92
Postoperative hospital stay (days), mean ± SD	5.59 ± 3.26
Complications, n (%)	4 (10.81%)
Hemorrhage	1 (2.70%)
Cholangitis	1 (2.70%)
Pleural effusion	2 (5.41%)
Preoperative liver function	
ALT (U/L)	170.84 ± 114.65
AST (U/L)	146.30 ± 115.29
TBIL (μmol/L)	93.78 ± 55.94
DBIL (μmol/L)	63.73 ± 42.05
Liver function 3 days postoperatively	
ALT (U/L)	73.65 ± 38.85
AST (U/L)	61.05 ± 37.07
TBIL (μmol/L)	76.05 ± 33.90
DBIL (μmol/L)	49.32 ± 25.93

### Follow-up outcome

All patients were reviewed for residual stones by the abdominal US or CT at 7 days postoperatively. The initial clearance rate of stones was 40.54% (15/37), and the final clearance rate of stones was 100% (37/37) after subsequent rigid choledochoscopic lithotripsy. During a mean follow-up period of 23 months (range 12 to 36 months), there were no patient deaths and only one patient experienced stone recurrence (Table 3).

**Table 3:** Follow-up outcome

Variables	Value (*n* = 37)
Initial stone clearance, yes. (%)	15 (40.54%)
Final stone clearance, yes. (%)	57 (100.00%)
Follow-up periods (month), mean ± SD	23.32 ± 7.57
Stone recurrence, yes. (%)	1 (2.70%)
Residual biliary Stricture	1 (2.70%)

## Discussion

RHL is highly prevalent in East and Southeast Asia, with an incidence rate of about 4–24%, among which, the incidence rate of HL in China ranks first in the world, which is about 24% [5, 6, 15]. RHL is prone to further exacerbate the bile duct obstruction if it is not treated in time, which may lead to cholangitis, liver abscess, and even liver failure [16, 17]. Related reports show that 3.7–14.1% of HL eventually develop biliary cirrhosis and 3.3–21.2% develop HL-related intrahepatic cholangiocellular carcinoma [18]. In our study, preoperative comorbid hepatic tumours were excluded, and no hepatic tumours were detected in patients during a mean follow-up period of 23 months.

The treatment of RHL is currently still surgical, and the main treatment modalities include hepatectomy, PTCS, choledochojejunostomy, endoscopic lithotripsy, with the aim of removing stones, relieving stenosis, and patrolling drainage [19, 20]. Among them, hepatectomy is considered the first choice for the treatment of HL, but in patients who have a wide distribution of stones, partial hepatectomy is limited, and it is often required to be combined with other surgical procedures [3, 18]. Li performed hepatectomy combined with cholangioscopy for stone extraction in 56 patients with HL, stone clearance and recurrence rates were 92.9% and 13.5%, and 26.8% of patients experienced postoperative complications [21]. Endoscopic retrograde cholangiopancreatography (ERCP) is considered safe and effective for stones located below grade II bile ducts, but ERCP disrupts the structure of the sphincter of Oddi, and intestinal contents and bacteria are prone to retrograde into the bile ducts, which can lead to recurrence of stones [5, 22]. ERCP treatment of stones located in grade III and IV bile ducts as well as larger stones is difficult. In addition, ERCP is considered difficult in patients with RHL who have bile duct reconstruction, and the literature shows a 16% stone retention rate and an 8–12% complication rate after ERCP for CBD [22, 23].

As a result of multiple previous biliary surgeries, the probability of abdominal adhesions, changes in biliary and vascular structures, and insufficient hepatic functional reserve is greatly increased in patients with RHL. This situation undoubtedly increases the difficulty of reoperation, which is a challenge for the operator and can greatly affect the outcome.

PTCS was first proposed by Nimura in the 1980s, which establishes a channel between the bile duct and the outside of the body through a one-stage puncture, and gradually expands this channel at a later stage until the choledochoscope is accessible, thus facilitating choledochoscopic access to the bile ducts for stone extraction [11, 24]. For patients of advanced age, those who refuse major surgery or cannot tolerate major surgery, especially those whose stones are distributed in multiple segments of the liver and those with RHL, PTCS has obvious advantages in terms of the scope of adaptation of the procedure, the size of the trauma, the reproducibility of the procedure, the therapeutic efficacy, and the postoperative recovery [25]. Wang used PTCS to treat 118 cases of HL and achieved good therapeutic results, with final stone clearance and recurrence rates of 78.8% and 14.4%, respectively, and a complication rate of 7.6% [14].

However, PTCS also suffers from the problems of a high number of surgeries, the need for multiple sinus dilatation, and the long treatment period. Some scholars proposed PTOBF based on traditional PTCS, which simplifies the operation of stepwise sinus dilation by dilating the sinus to 16Fr at one time after a successful puncture, and then directly performing choledochoscopic exploration and stone extraction [14, 26]. Relevant reports have shown that compared with conventional PTCS, PTOBF combined with rigid cholangioscopy can perform cholangiostomy and stone extraction in a single operation, which significantly shortens the treatment period, and also relieves intrahepatic bile duct stenosis, as well as provides better stone removal results and postoperative recovery time, which has been gradually applied in China [14]. Previous studies have shown that US guidance significantly reduces the complications and radiation dose of biliary puncture with high safety and reliability [27, 28].

All patients in this study were treated with US-guided PTOBF combined with rigid choledochoscopy, and real-time US navigation throughout the procedure improved the success rate of puncture and also helped us to find the location of the stones, which in turn improved the rate of stone removal. Intraoperative US-guided puncture can help us understand the anatomy of the bile ducts as well as the vascular system, which is more conducive to the puncture needle to accurately reach the target bile ducts, avoiding blood vessels and reducing bleeding under blind puncture.

In addition, most of our lithotripsy operations were carried out in the sheath, which avoided accidental injury to the bile ducts during the operation, and at the same time, the sheath was also able to exert a certain degree of pressure on the sinusoids to stop bleeding, which further reduced the surgical complications. In addition, bile duct stenosis is a major risk factor for stone recurrence after HL surgery [29]. The postoperative placement of a 16 Fr biliary drainage catheter in the patients in this study helped to dilate the narrowed bile ducts, thereby reducing the rate of stone recurrence. In this study, the final stone clearance rate was 100%, the postoperative complication rate was 10.81%, and the stone recurrence rate was 2.70% after a median follow-up of 23 months, and our outcomes were superior to those previously reported using other surgical approaches [21, 25, 30]. Cheon followed 236 patients with HL who were treated with hepatectomy (*n* = 90), PTCS (*n* = 97), or ERCP (*n* = 49), with stone clearance rates of 83%, 64%, and 43%, stone recurrence rates of 18%, 21%, and 25%, and 63.9%, complications in 3% of the patients with PTCS, and surgery-related death in 1 patient with hepatectomy [25]. Compared with previous studies, US-guided PTOBF combined with rigid choledochoscopy is safe and feasible for the treatment of RHL and also achieves satisfactory clinical results.

**Fig. 2:**
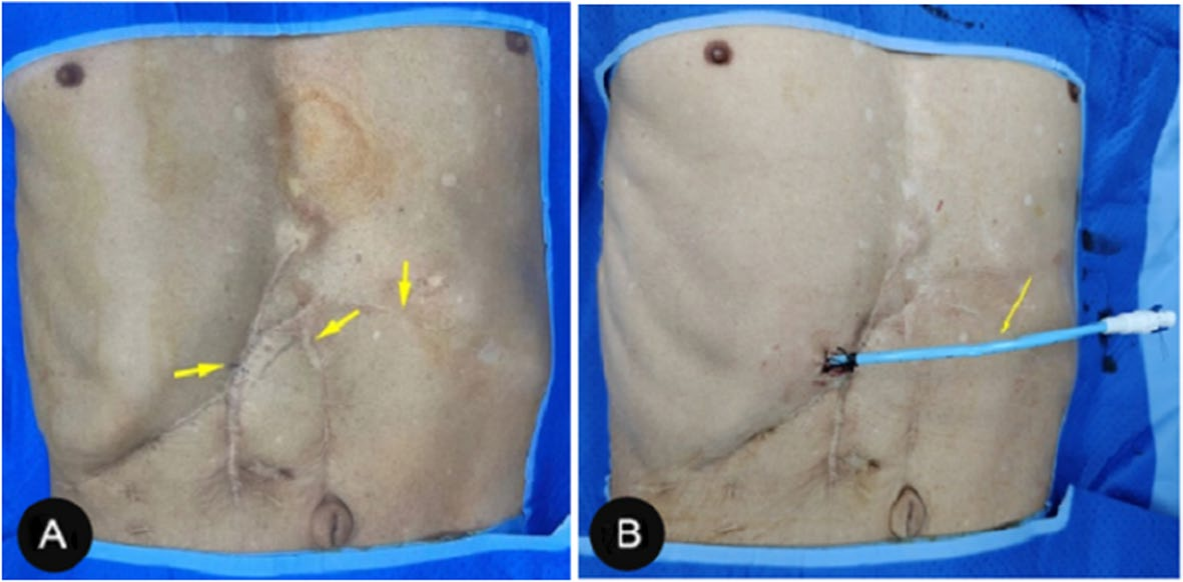
Comparison before and after PTOBF surgery. **A** Previous surgical scarring. **B** The PTOBF surgical incision is only a small incision

In the past, reoperation in RHL patients with a history of multiple biliary tract surgeries was very difficult. Many patients have extremely severe abdominal adhesions due to the multiple surgeries they have undergone, which, together with prolonged reoperation, insufficient residual liver function, poor incision healing, and high trauma often lead to unsatisfactory surgical results and greater physical and psychological trauma to the patients [31]. For patients with RHL, the liver is more fixed in position due to the adhesion of the liver to the surrounding organs created by previous surgeries and is instead more suitable for PTOBF treatment. US-guided PTOBF combined with rigid choledochoscopy is performed through only one or several small incisions, which is an obvious advantage over other methods of treating RHL (Fig. 2). In addition, PTOBF has a wide range of indications; one patient in this study had undergone three hepatectomies as well as two cholangiojejunostomy surgeries, and the stones recurred again after surgery; this patient’s condition did not support further surgery, and we treated him with US-guided PTOBF combined with rigid cholangioscopy, and finally this patient recovered and was discharged from the hospital. We followed him up for 25 months and did not see any stone retention or recurrence. Our approach may be a suitable option for RHL patients who refuse surgery because they cannot physically tolerate other surgical blows.

### Limitations and prospects

This study is retrospective and lacks a comparison group; long-term prospective randomized controlled trials with larger sample sizes are needed to confirm the safety as well as the reliability of PTOBF. In addition, the success of PTOBF is largely dependent on the experience and skill of the surgeon, and the results may vary greatly between surgical teams. Based on our previous successful experience, our center is ready to launch PTOBF for Child–Pugh C HL patients without coagulation disorders, and gradually broaden the range of indications for PTOBF. We believe that the application of PTOBF for the treatment of diseases within the biliary system will be very promising.

## Conclusion

US-guided PTOBF combined with rigid choledochoscopy for RHL has relatively good stone extraction results, a short hospital stay, and a certain therapeutic effect on bile duct stenosis, which may be a minimally invasive, safe, and effective treatment for RHL. However, this procedure relies on the surgeon’s proficient operative skills, and its therapeutic efficacy remains to be confirmed by comparative studies with larger sample sizes.

Better stone removal, shorter hospitalization time, and also has a therapeutic effect on bile duct stenosis, which is a minimally invasive, safe, and effective method.

## Data Availability

No datasets were generated or analysed during the current study.

## References

[1] Kim HJ, Kim JS, Joo MK, Lee BJ, Kim JH, Yeon JE, Park JJ, Byun KS, Bak YT (2015). Hepatolithiasis and intrahepatic cholangiocarcinoma: a review. World J Gastroenterol.

[2] Feng X, Zheng S, Xia F, Ma K, Wang S, Bie P, Dong J (2012). Classification and management of hepatolithiasis: a high-volume, single-center’s experience. Intractable Rare Dis Res.

[3] Dilek ON, Atasever A, Acar N, Karasu S, Ozlem Gur E, Ozsay O, Camyar H, Dilek FH (2020). Hepatolithiasis: clinical series, review and current management strategy. Turk J Surg.

[4] Ran X, Yin B, Ma B (2017). Four major factors contributing to intrahepatic stones. Gastroenterol Res Pract.

[5] Zhou B, Hu J, Zhong Y (2017). Surgical treatments for patients with recurrent bile duct stones and Oddis sphincter laxity. Intractable Rare Dis Res.

[6] Oh CH, Dong SH (2015). Recent advances in the management of recurrent bile duct stones. Korean J Gastroenterol.

[7] Yang T, Lau WY, Lai EC, Yang LQ, Zhang J, Yang GS, Lu JH, Wu MC (2010). Hepatectomy for bilateral primary hepatolithiasis: a cohort study. Ann Surg.

[8] Cui L, Xu Z, Ling XF, Wang LX, Hou CS, Wang G, Zhou XS (2014). Laparoscopic hepaticoplasty using gallbladder as a subcutaneous tunnel for hepatolithiasis. World J Gastroenterol.

[9] Lorio E, Patel P, Rosenkranz L, Patel S, Sayana H (2020). Management of hepatolithiasis: review of the literature. Curr Gastroenterol Rep.

[10] Li C, Wen T (2017). Surgical management of hepatolithiasis: a minireview. Intractable Rare Dis Res.

[11] Nimura Y, Shionoya S, Hayakawa N, Kamiya J, Kondo S, Yasui A (1988). Value of percutaneous transhepatic cholangioscopy (PTCS). Surg Endosc.

[12] Tao H, Wang P, Sun B, Li K, Zhu C (2020). One-step multichannel percutaneous transhepatic cholangioscopic lithotripsy applied in bilateral hepatolithiasis. World J Surg.

[13] Neuhaus H (1999). Intrahepatic stones: the percutaneous approach. Can J Gastroenterol.

[14] Wang P, Sun B, Huang B, Xie J, Liu Y, Zhu C, Ye C, Zhou Z (2016). Comparison between percutaneous transhepatic rigid cholangioscopic lithotripsy and conventional percutaneous transhepatic cholangioscopic surgery for hepatolithiasis treatment. Surg Laparosc Endosc Percutan Tech.

[15] Sugiyama M, Atomi Y (2002). Risk factors predictive of late complications after endoscopic sphincterotomy for bile duct stones: long-term (more than 10 years) follow-up study. Am J Gastroenterol.

[16] Fan WJ, Zou XJ (2022). Subacute liver and respiratory failure after segmental hepatectomy for complicated hepatolithiasis with secondary biliary cirrhosis: a case report. World J Gastrointest Surg.

[17] Liang T, Su W, Zhang Q, Li G, Gao S, Lou J, Zhang Y, Ma T, Bai X (2016). Roles of sphincter of oddi Laxity in bile duct microenvironment in patients with cholangiolithiasis: from the perspective of the microbiome and metabolome. J Am Coll Surg.

[18] Xia H, Zhang H, Xin X, Liang B, Yang T, Liu Y, Wang J, Meng X (2023). Surgical management of recurrence of primary intrahepatic bile duct stones. Can J Gastroenterol Hepatol.

[19] Tazuma S, Unno M, Igarashi Y, Inui K, Uchiyama K, Kai M, Tsuyuguchi T, Maguchi H, Mori T, Yamaguchi K, Ryozawa S, Nimura Y, Fujita N, Kubota K, Shoda J, Tabata M, Mine T, Sugano K, Watanabe M, Shimosegawa T (2017). Evidence-based clinical practice guidelines for cholelithiasis 2016. J Gastroenterol.

[20] Huang ZQ, Huang XQ, Zhang WZ, Xu LN, Yang T, Zhang AQ, Dong JH (2008). Liver resection in hepatolithiasis: 20-year’s evolution. Zhonghua Wai Ke Za Zhi.

[21] Li EL, Yuan RF, Liao WJ, Feng Q, Lei J, Yin XB, Wu LQ, Shao JH (2019). Intrahepatic bile duct exploration lithotomy is a useful adjunctive hepatectomy method for bilateral primary hepatolithiasis: an eight-year experience at a single centre. BMC Surg.

[22] Cianci P, Restini E (2021). Management of cholelithiasis with choledocholithiasis: Endoscopic and surgical approaches. World J Gastroenterol.

[23] Dollhopf M, Schmetkamp H (2022). Endoscopic management of difficult common bile duct stones. Minerva Gastroenterol (Torino).

[24] Nimura Y, Kamiya J, Hayakawa N, Shionoya S (1989). Cholangioscopic differentiation of biliary strictures and polyps. Endoscopy.

[25] Cheon YK, Cho YD, Moon JH, Lee JS, Shim CS (2009). Evaluation of long-term results and recurrent factors after operative and nonoperative treatment for hepatolithiasis. Surgery.

[26] Qin J, He Y, Ma L, Duan J, Duan R, Liu R, Zhou J, Yang N, Li Y, Xiong Y, Li H, Zeng X, Li C, Li X (2023). Efficacy of 3D-printed assisted percutaneous transhepatic one-step biliary fistulation combined with rigid choledochoscopy for intrahepatic bile duct stones. Dig Liver Dis.

[27] Giurazza F, Corvino F, Contegiacomo A, Marra P, Lucarelli NM, Calandri M, Silvestre M, Corvino A, Lucatelli P, De Cobelli F, Niola R, Cariati M, Italian College of Interventional Radiology Rising Stars, G (2019). Safety and effectiveness of ultrasound-guided percutaneous transhepatic biliary drainage: a multicenter experience. J Ultrasound.

[28] Park SE, Nam IC, Baek HJ, Ryu KH, Lim SG, Won JH, Kim DR (2022). Effectiveness of ultrasound-guided percutaneous transhepatic biliary drainage to reduce radiation exposure: a single-center experience. PLoS One.

[29] Jan YY, Chen MF (1995). Percutaneous trans-hepatic cholangioscopic lithotomy for hepatolithiasis: long-term results. Gastrointest Endosc.

[30] Park JS, Jeong S, Lee DH, Bang BW, Lee JI, Lee JW, Kwon KS, Kim HK, Shin YW, Kim YS, Park SG (2013). Risk factors for long-term outcomes after initial treatment in hepatolithiasis. J Korean Med Sci.

[31] Pu T, Chen JM, Li ZH, Jiang D, Guo Q, Li AQ, Cai M, Chen ZX, Xie K, Zhao YJ, Wang C, Hou H, Lu Z, Geng XP, Liu FB (2022). Clinical online nomogram for predicting prognosis in recurrent hepatolithiasis after biliary surgery: a multicenter, retrospective study. World J Gastroenterol.

